# A Novel MRI Compatible Balance Simulator to Detect Postural Instability in Parkinson's Disease

**DOI:** 10.3389/fneur.2019.00922

**Published:** 2019-08-28

**Authors:** Elizabeth P. Pasman, Martin J. McKeown, Taylor W. Cleworth, Bastiaan R. Bloem, J. Timothy Inglis, Mark G. Carpenter

**Affiliations:** ^1^School of Kinesiology, University of British Columbia, Vancouver, BC, Canada; ^2^Pacific Parkinson's Research Centre, Djavad Mowafaghian Centre for Brain Health, University of British Columbia and Vancouver Coastal Health, Vancouver, BC, Canada; ^3^School of Kinesiology and Health Science, York University, Toronto, ON, Canada; ^4^Department of Neurology, Donders Institute for Brain, Cognition and Behaviour, Radboud University Medical Center, Nijmegen, Netherlands; ^5^Djavad Mowafaghian Centre for Brain Health, University of British Columbia, Vancouver, BC, Canada; ^6^International Collaboration on Repair Discoveries, University of British Columbia, Vancouver, BC, Canada

**Keywords:** Parkinson's disease, postural instability, elderly, static balance, dynamic balance, balance simulator, center of mass sway, kinematics

## Abstract

**Background:** Postural instability is a debilitating and largely treatment-resistant symptom of Parkinson's disease (PD). A better understanding of the neural substrates contributing to postural instability could lead to new targets for improved pharmacological and neurosurgical interventions. However, investigating these neural substrates necessitates the use of functional MRI scanners, which are almost exclusively horizontally-based.

**Objective:** We aimed to develop, and validate the use of, an MRI compatible balance simulator to study static and dynamic balance control in PD patients and elderly controls.

**Methods:** Our MRI compatible balance simulator allowed participants to actively balance an inverted pendulum by activating postural muscles around the ankle joint while supine. Two studies were performed to compare static and dynamic balance performance between upright stance and simulated stance in PD patients and controls. Study 1 (14 PD; 20 controls) required participants to maintain static balance during upright and simulated stance for 120 s with eyes open and closed. In study 2 (20 PD; 22 controls) participants repeated the static balance task (80 s, eyes closed only), and also completed a dynamic balance task which required maintaining balance while experiencing random anterior-posterior perturbations applied to the trunk/pendulum. Postural sway of the body/pendulum was measured using an angular velocity sensor (SwayStar^TM^, study 1) and Optotrak motion capture (study 2). Outcome measures were amplitude and frequency of center of mass sway for static balance, and peak and time-to-peak of center of mass displacement and velocity for dynamic balance.

**Results:** PD patients had larger sway amplitude during both upright and simulated static balance compared to controls. PD patients had larger peak and time-to-peak sway, and larger time-to-peak sway velocity, during simulated, but not upright, dynamic balance compared to controls.

**Conclusions:** Deficits in static and dynamic balance control can be detected in PD patients using a novel MRI compatible balance simulator. This technique allows for functional neuroimaging to be combined with balance-relevant tasks, and provides a new means to create insights into the neural substrates contributing to postural instability in PD.

## Introduction

Postural instability and falls are common in Parkinson's disease (PD) patients, resulting in significant disability, loss of independence, and reduced quality of life (1). Pharmacological and neurosurgical treatments currently used for PD are unable to alleviate, and may in some cases even aggravate, postural instability (2–4). In addition, since the pathophysiology underlying postural instability is insufficiently understood (1, 3, 4), a better understanding of the neural substrates contributing to postural instability in PD could lead to new targets for improved pharmacological and neurosurgical interventions.

PD patients have unique balance deficits. Current evidence suggests PD patients exhibit larger angular and linear displacement and velocity of the trunk during static balance compared to elderly controls (5, 6). While vision affects sway amplitude and frequency (7, 8), the effects of PD on static balance performance are independent of visual condition (9–11). Additionally, in response to dynamic perturbations delivered to the trunk, PD patients show increased trunk displacement compared to elderly controls (12).

There would be tremendous benefit in being able to investigate the network of cortical and subcortical structures contributing to postural control normally and in PD. However, this necessitates the use of functional MRI scanners, which are almost exclusively horizontally-based. While some studies have had participants perform balance-related tasks while lying down, none of the tasks involved participants actively maintaining equilibrium of a free-standing balance system (13–15). Motor imagery may offer an alternative for complex upright tasks such as walking (16, 17), but may be limited when studying non-volitional sensorimotor tasks such as static and reactive dynamic balance. Also, although motor imagery and motor execution of the same task share neural substrates, subtle but important differences exist (18).

The aim of the current study was to develop, and validate the effectiveness of using, an MRI-compatible balance simulator to investigate static and dynamic balance control in PD patients and elderly controls, in tasks commonly used to identify balance deficits (19). For the balance simulator to be effective, it should be relatively easy for both healthy participants and PD patients to control the simulator after only a few minutes of practice. Moreover, the simulator should elicit balance behaviors comparable to those observed during upright balancing tasks and be sensitive to PD changes seen in both static and dynamic balance performance.

We hypothesized that PD patients, compared to elderly controls, would show larger amplitude and higher frequency of sway during static balancing, independent of vision, and increased peak sway during dynamic balancing, when standing upright, as well as when actively controlling the balance simulator. To test these hypotheses, we performed two independent studies: the first investigated only static balancing; and the second investigated both static and dynamic balancing. These studies serve to validate the novel balance simulator for future use in functional MRI experiments.

## Materials and Methods

### Participants

Eighty-five participants (38 PD patients, 47 elderly controls) participated across two independent studies. Exclusion criteria for PD patients were any of the following medical issues (self-reported during initial screening): any prior neurosurgical procedures such as deep brain stimulation; excessive levodopa-induced dyskinesia that impaired their balance; botulinum toxin injections in lower leg muscles within the last 3 months; documented proprioceptive loss (e.g., abnormal vibratory sense, altered joint position sense, etc.); dementia precluding informed consent; history of other neurological disease (e.g., stroke, seizures); and medical issues (other than PD) that influenced their balance. Exclusion criteria for controls were any medical issues (self-reported during initial screening) that influenced their balance, including ankle injuries/surgery, stroke, conditions affecting vestibular function, diabetes, and conditions resulting in a loss of sensation in the feet and/or lower legs. All participants were fluent in English, provided written informed consent prior to testing, and followed experimental procedures that were approved by the UBC Clinical Research Ethics Board. Nine participants were excluded due to: technical issues (*n* = 5), inability to complete the protocol (*n* = 3), or withdrawal of consent due to anxiety (*n* = 1). As a result, study 1 included 34 participants (14 PD patients; 20 controls) and study 2 included 42 participants (20 PD patients; 22 controls; see Table 1 for details).

**Table 1:** Baseline participant characteristics for study 1 and study 2.

	**Study 1**			**Study 2**		
	**PD_ON_**	**Controls**	***p*-value**	**PD_ON_**	**Controls**	***p*-value**
**GENERAL INFORMATION**						
Sample size	14	20		20	22	
Age (years)	69.0 (1.6)	68.3 (1.4)	0.734	67.6 (1.0)	68.4 (1.2)	0.581
Number of women (%)	6 (43%)	11 (55%)	0.728	9 (45%)	13 (59%)	0.537
Height (cm)	173.3 (2.8)	169.4 (1.6)	0.200	168.6 (1.5)	166.5 (1.9)	0.409
Weight (kg)	72.4 (3.9)	70.6 (3.4)	0.742	71.9 (3.0)	66.0 (2.9)	0.168

*Data are displayed as mean (SE) or number of persons (percentage between parentheses); PD_ON_, Parkinson's disease patients*.

All PD patients were examined ~1 h after intake of their regular antiparkinson medication to coincide with their subjectively best clinical “on” condition (PD_ON_). We purposely opted for this condition for two reasons: first, balance control is usually not altered much by dopaminergic medication; and second, testing PD_ON_ patients avoids any potential confounds of fatigue, anxiety, and cumbersome bradykinesia/rigidity that may accompany the “off” phase. All participants completed a brief medical history survey. PD_ON_ patients were clinically assessed using the Hoehn and Yahr (H&Y) scale (20) and Unified Parkinson's Disease Rating Scale motor examination (UPDRS-ME) (21) (Appendix 1).

### Apparatus

**Figure 1:**
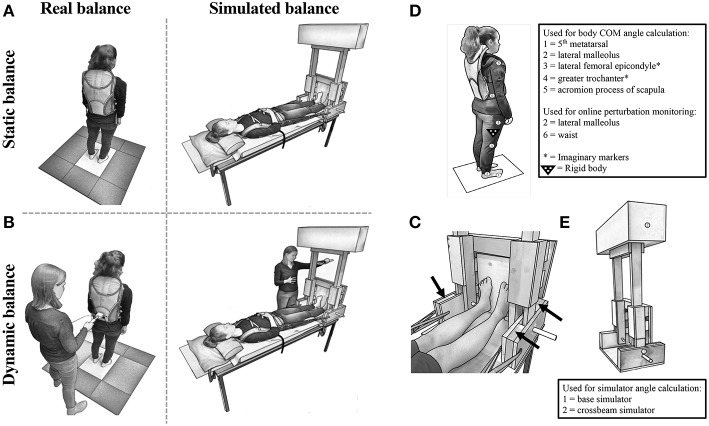
Experimental set-up for **(A)** Static_Real_ and Static_Sim_ (Note: spotter was present but is not shown here), and **(B)** Dyn_Real_ and Dyn_Sim_. **(C)** Close-up of the balance simulator with black arrows indicating mechanical stops. Kinematic marker set-up for **(D)** real and **(E)** simulated balance.

Simulated stance trials were performed using a customized balance simulator (“simulator”) made completely from non-ferrous MRI compatible material (wood, glue, and plastic/glass bearings). In the simulator, the participant lay supine with their feet placed at a width equal to their foot length against a footplate that rotated about an axis aligned with the ankle joints that controlled a free-standing inverted pendulum (Figures 1A–C). The center of mass (COM) of the balance-arm of the simulator was located ~1 m above the axis of rotation. For each participant individually, the ankle load stiffness seen during normal upright quiet stance was calculated using the formula:

$$\begin{array}{l} S=m\cdot g\cdot h, \end{array}$$

where *S* = load stiffness, *m* = body mass (kg), *g* = gravitational acceleration constant (9.81 m/s^2^), and *h* = height of the participant's estimated COM (m) (22). The mass on the simulator was adjusted to achieve ~75% of the ankle load stiffness estimated during upright quiet stance for study 1 (range = 54–87%, mean ± SD = 76 ± 8.1%); and 60% for study 2 (38–81%, 60 ± 8.4%). The current studies served to validate the simulator for future use in functional MRI experiments. MRI-scanning tables typically have a table weight limit; which was 300 lbs. for the MRI-scanner available at our research institution at UBC. Therefore, the weight that could be added to the simulator to increase load stiffness was limited by the total weight of the participant and simulator combined. To prevent participants from being pushed away from the footplate when balancing the simulator, and eliminate corresponding movements of the trunk and head, tightly-fastened adjustable straps wrapped around the waist and shoulders were attached to the base of the simulator and tightened to maximum level tolerated by the participant. Mechanical stops were used to limit the simulator to a range of +/– 17° to ensure participant safety (Figure 1C).

### Experimental Protocol for Study 1

Participants performed a series of real static balancing tasks (Static_Real_) while standing upright and simulated static balancing tasks (Static_Sim_) while lying down using the simulator (Figure 1A). In both conditions, two practice trials of at least 60 s [1 eyes open (EO) and 1 eyes closed (EC)] were performed first to allow participants to become familiar with the procedures, visual feedback, and to remove any potential first trial effects (23). Participants then performed two additional EO and EC trials in alternating order with a total duration of 120 s each (8, 24). Trials were separated by a few minutes of rest. The order of the balance and visual conditions were counterbalanced across participants. A spotter stood next to the participants to assist them in case there was a loss of balance in both balance conditions.

During EO Static_Sim_ trials, participants were provided with real-time visual feedback of the simulator. A potentiometer, attached to one side of the axis about which the simulator rotated, was calibrated and used to determine the angular position of the simulator. The angular position was then used to render a visual scene (Vizard, WorldViz, USA) displayed on a monitor located 0.4 m in front of the participant.

In the Static_Real_ condition, participants stood quietly with their arms hanging loosely by their sides, and feet placed at a width equal to their foot length during each trial. Foot position was marked to ensure that participants returned to the same foot position in every trial. Participants were instructed to stand as still as possible.

At the start of each Static_Sim_ trial, the experimenter positioned the simulator ~3° from vertical, leaning toward the participant, to mimic upright stance (25). Participants were given verbal feedback by the experimenter until they fully controlled the simulator and were then required to keep the simulator as still as possible for the duration of the trial.

### Experimental Protocol for Study 2

Participants performed two Static_Real_ and Static_Sim_ trials using a similar protocol to study 1. Trials were 80 s in duration, and performed only with EC, as PD effects on Static_Sim_ performance were found to be independent of vision in study 1 (see results). Participants also performed dynamic balancing tasks, in which they were required to maintain balance while responding to repetitive perturbations applied to the COM (a sudden push delivered to the participant's lower back or the simulator by the experimenter), when standing upright (Dyn_Real_) and in the simulator (Dyn_Sim_) (Figure 1B). Specifically, participants were instructed to respond to perturbations with corrections about the ankle joint without moving their feet. In both Dyn_Real_ and Dyn_Sim_, perturbations were delivered in the anterior-posterior (AP) plane by the experimenter using a hand-held bar, instrumented with a load transducer (sampled at 1 kHz) to control perturbation force. In addition, online calculation of waist or simulator angular displacement provided monitoring of the perturbation magnitude. Most perturbations were in the forward direction, with some backward perturbations (a sudden backward pull to the lower back or simulator) serving as catch trials to prevent anticipatory leaning (percentage of catch trials for Dyn_Real_: range = 8–23%, mean ± SD = 15 ± 3.8%; Dyn_Sim_: 6–22%, 12 ± 4.2%). The inter-perturbation interval ranged from 2.0 to 8.4 s for Dyn_Real_ and from 2.5 to 28.2 s for Dyn_Sim_. The order of the balance condition and task were counterbalanced across participants.

### Measurements for Study 1

Postural sway in the AP direction was measured using an angular velocity sensor (SwayStar^TM^, Balance Int. Innovations GmbH, Switzerland), mounted on the participants' trunk at the level of the lower back (L1–L3) near the body's COM (Static_Real_) or on the crossbeam of the simulator (Static_Sim_). Angular velocity signals were sampled at 100 Hz and used to calculate angular displacement via trapezoidal integration. The angular displacement signals were low-pass filtered offline using a fourth-order, 3.5 Hz cutoff dual-pass Butterworth filter, to remove rest and postural tremors in PD patients which have a typical frequency between 4 and 7 Hz (26). Data were clipped at 80 s to coincide with the longest duration that all participants could balance the simulator across trials, and still meet the recommended minimum of 60 s for stance trials (8, 24). The mean was removed from the signal prior to calculating root mean square (RMS) and mean power of frequency (MPF), which were averaged over the 2 trials for EO and EC conditions.

### Measurements for Study 2

Postural sway was measured using an OPTOTRAK (NDI, Waterloo, Canada) motion capture system (sampled at 125 Hz). The placement of infrared markers and rigid bodies are illustrated in Figures 1D,E. Missing data (< 40 ms) was interpolated using cubic spline. Kinematic data were low-pass filtered offline using a fourth order dual-pass Butterworth filter with either a 3.5 Hz (Static) or 5 Hz (Dyn) cutoff. Total body COM displacement was calculated for Static_Real_ and Dyn_Real_ trials using a four-segment model from 2-dimensional filtered coordinates defining the foot, shank, thigh, and head/arms/trunk segments (27) in conjunction with anthropometric data (28). For both Static_Real_ and Dyn_Real_ trials, AP angular COM displacement was calculated using the inverse tangent function.

For Static_Real_ and Static_Sim_ trials, the mean was removed from the COM signal prior to calculating the RMS and MPF, which were averaged over both trials. Static_Real_ total body COM data were unavailable for one participant due to technical difficulties during collection.

For Dyn_Real_ and Dyn_Sim_ trials, mean force + 4 SD was calculated offline from 500 ms pre-perturbation and used as a threshold to detect perturbation onset. The area under the curve from perturbation onset to the first zero crossing was calculated. Individual perturbations were excluded if the perturbation force exceeded the mean ± 1 SD range of the perturbation force calculated across all perturbations and participants within the Dyn_Real_ and Dyn_Sim_ conditions. Remaining trials were used to calculate peak and time-to-peak AP angular COM displacement and velocity within each condition and participant. A minimum of 5 perturbations was required for the participant's data to be included in the final analysis. Data from 5 participants were removed due to: an inability to successfully complete the Dyn_Sim_ trials (*n* = 2), or technical difficulties during collection (*n* = 3).

### Statistical Analysis

Assumptions of normality were validated using Shapiro-Wilk's test and inspection of histograms and quantile-quantile plots. Baseline characteristics between PD_ON_ patients and controls were compared using independent *t*-tests, Mann-Whitney tests, or Chi-square tests where appropriate.

For study 1, all dependent measures were analyzed using a 2 × 2 mixed design analysis of variance with group (PD_ON_, controls) and vision (EO, EC) as independent variables for Static_Real_ and Static_Sim_ separately. Non-normal data were log-transformed prior to analysis. For all dependent measures, Levene's tests demonstrated equality of variances across groups, and Box M's tests demonstrated equality of covariance matrices. Partial eta-squared was used to assess effect size.

For study 2, all dependent measures were compared between PD_ON_ patients and controls for Static and Dyn tasks separately using independent *t*-tests and Mann-Whitney tests where appropriate. Cohen's *d* and eta-squared were used to assess effect size where appropriate.

An overall α < 0.05 was used for all statistical comparisons. Unless otherwise stated, results are means ± SE.

## Results

### Static Balancing Tasks

**Figure 2:**
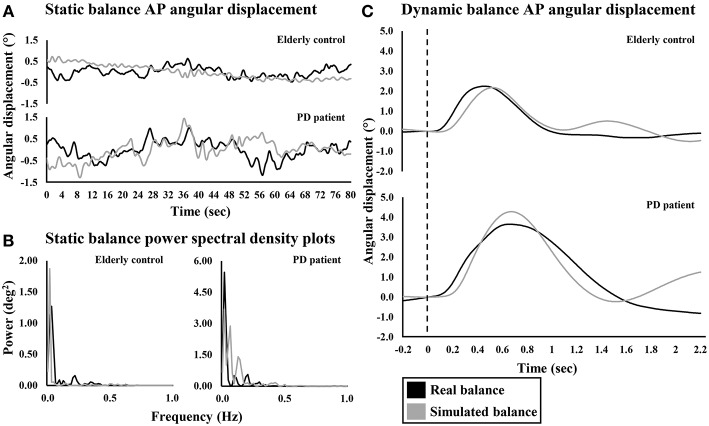
Representative anterior-posterior angular displacement traces and power spectral density plots of an elderly control and PD patient during real and simulated **(A,B)** static and **(C)** dynamic balance. Dashed line indicates perturbation onset for dynamic balance.

**Figure 3:**
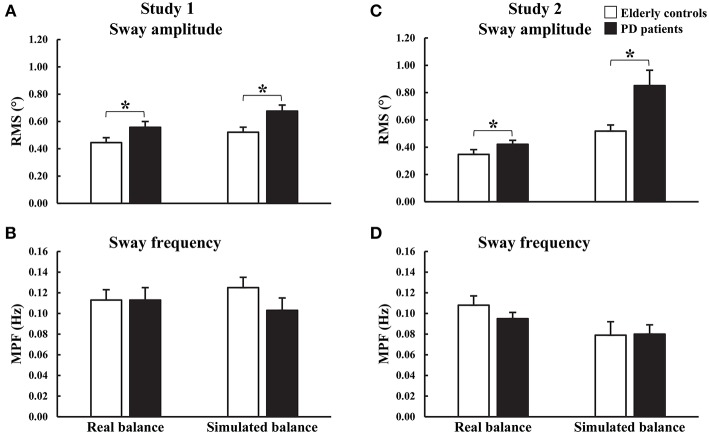
Group means and standard errors of **(A,C)** root mean square (RMS) and **(B,D)** mean power of frequency (MPF) of angular displacements during real and simulated static balance. Study 1: eyes open and eyes closed data were combined. *p < 0.05.

During both studies, differences in upright quiet standing performance were observed between PD_ON_ patients and controls (Figures 2A,B, 3 and Appendix 2). During study 1, there was a significant main effect of group on trunk AP RMS [*F*_(1, 32)_ = 4.725, *p* = 0.037] with significantly larger displacements in PD_ON_ patients (0.557 ± 0.043°) than controls (0.445 ± 0.036°). There was a significant main effect of vision on trunk AP RMS [*F*_(1, 32)_ = 11.674, *p* = 0.002] with significantly larger displacements in EC trials (0.539 ± 0.032°) than EO trials (0.462 ± 0.030°). No significant main effects for trunk AP MPF, or interactions for trunk AP RMS or MPF, were found. During study 2, there was a significant main effect of group on COM AP RMS (*U* = 132.0, *z* = −2.034, *p* = 0.042) with significantly larger displacements in PD_ON_ patients (median = 0.438°) than controls (median = 0.331°). There was no significant main effect of group on COM AP MPF.

During both studies, differences in simulated static balance performance were observed between PD_ON_ patients and controls (Figures 2A,B, 3 and Appendix 2). Similar to upright quiet standing, during study 1 there was a significant main effect of group on simulator AP RMS [*F*_(1, 32)_ = 7.205, *p* = 0.011] with significantly larger displacements in PD_ON_ patients (0.676 ± 0.044°) than controls (0.521 ± 0.037°). There was also a significant main effect of vision on simulator AP MPF [*F*_(1, 32)_ = 5.143, *p* = 0.030] with significantly higher MPF in EC trials (0.121 ± 0.008°) than EO trials (0.107 ± 0.010°). No significant interactions were found for simulator AP RMS or MPF. During study 2, there was a significant main effect of group on simulator AP RMS (*U* = 108.0, *z* = −2.660, *p* = 0.007] with significantly larger displacements in PD_ON_ patients (median = 0.738°) than controls (median = 0.500°). There was no significant main effect of group on simulator AP MPF.

### Dynamic Balancing Tasks

Perturbation force was not significantly different between the PD_ON_ patients (Dyn_Real_: 15.491 ± 0.537 N; Dyn_Sim_: 4.310 ± 0.107 N) and controls (Dyn_Real_: 16.244 ± 0.528 N; Dyn_Sim_: 4.025 ± 0.151 N).

**Figure 4:**
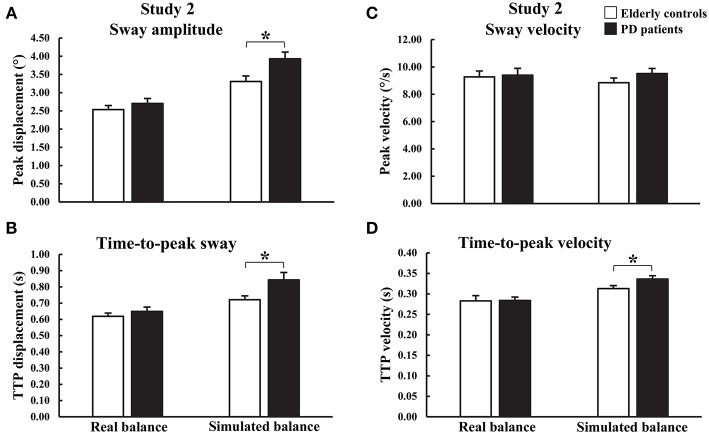
Group means and standard errors of peak and time-to-peak **(A,B)** amplitude and **(C,D)** velocity of angular displacements during real and simulated dynamic balance. *p < 0.05.

During Dyn_Real_, there were no significant main effects of group on COM peak angular displacement or velocity and COM time-to-peak angular displacement or velocity (Figures 2C, 4 and Appendix 2). In contrast, differences were observed between PD_ON_ patients and controls for Dyn_Sim_ (Figures 2C, 4 and Appendix 2). There was a significant main effect of group on simulator peak angular displacement (*U* = 55.0, *z* = −2.182, *p* = 0.029) with significantly larger displacement in PD_ON_ patients (median = 3.954°) than controls (median = 3.046°). There was a significant main effect of group on simulator time-to-peak angular displacement [*t*_(19.789)_ = −2.344, *p* = 0.030] with significantly longer time-to-peak in PD_ON_ patients (0.843 ± 0.046 s) than controls (0.721 ± 0.024 s). There was a significant main effect of group on simulator time-to-peak velocity (*U* = 34.0, *z* = −3.099, *p* = 0.001) with significantly longer time-to-peak velocity in PD_ON_ patients (median = 0.330 s) than in controls (median = 0.308 s). There was no significant main effect of group on simulator peak velocity.

## Discussion

The aim of this study was to develop, and validate the effectiveness of using, a novel MRI compatible balance simulator for investigating static and dynamic balance control in PD_ON_ patients and elderly controls. In order for the balance simulator to be effective, it should be relatively easy for both healthy participants and PD patients to control the simulator after only a few minutes of practice. Moreover, the simulator should elicit balance behaviors comparable to those observed during upright balancing tasks and be sensitive to PD changes seen in both static and dynamic balance performance.

The majority of participants that performed the protocol without technical issues were successful in completing the simulated balancing tasks with minimal practice (76/79 participants). The three participants unable to successfully complete the simulated balancing tasks failed to control the simulator for at least 80 s.

Sway characteristics recorded during simulated static balancing tasks were comparable to those observed during upright quiet standing (Figures 2A, 3). This suggests that a similar balance behavior is exhibited when maintaining balance using the simulator and when maintaining balance of the body during upright quiet standing.

During the Static_Real_ trials, PD_ON_ patients showed larger amplitude but no difference in frequency of AP sway compared to controls. These findings are consistent with previous work that investigated static balance control in PD_ON_ patients with similar disease severity and comparable sample durations and dependent measures (6). Congruent PD-related changes in sway were observed in the balance simulator, with significantly larger amplitude of AP sway compared to controls. Therefore, not only is the simulator able to elicit static balance behavior similar to that seen during upright quiet standing in the same participants, but differences in balance behavior between controls and PD_ON_ patients seen during real static balancing tasks can also be detected using the simulator.

During the Dyn_Real_ trials, no differences were found in peak and time-to-peak AP sway amplitude and velocity between PD_ON_ patients and controls. This conflicts with previous work reporting larger peak AP sway in response to a forward pull of the body at shoulder level in PD_ON_ patients with similar disease severity (H&Y stage of 1 or 2), albeit longer mean disease duration (8.3 ± 1.7 years vs. 5.6 ± 0.8 years in current study) (12). Although perturbation forces were similar across studies, there were several methodological differences such as the point of application of the perturbation on the torso, perturbation delivery method, and method of measuring peak sway. While no group differences were detected during Dyn_Real_ trials, larger peak amplitude, and longer time-to-peak amplitude and velocity were observed in the PD_ON_ patients compared to controls, despite similar perturbation forces, during the Dyn_Sim_ trials. Therefore, the simulator may be able to detect subtle differences in dynamic balance behavior between controls and PD_ON_ patients that are not observable during real dynamic balancing tasks.

While other studies have tried to develop systems allowing participants to perform balance-related tasks while lying down (13–15), none truly simulated free-standing balance. Karim et al. developed an MRI compatible force platform and had participants use visual feedback to control AP center of pressure movements generated by ankle dorsiflexor and plantarflexor activation (13). Participants used feed-forward volitional control to complete the task. De Lima-Pardini et al. developed an MRI compatible force measurement system designed to investigate anticipatory postural adjustments during single leg raises in order to simulate step initiation (14). Lastly, Buettner et al. developed a moveable balance board producing torque resembling gravity, inertia, and damping effects of free standing and asked participants to continuously balance the board by moving their feet in the ankle joint (15). While the balance board system took into account the characteristics of an inverted pendulum body, participants did not balance a physical weight but instead a torque was generated using an electric motor with the potential effect of electromechanical delays limiting the results of the study. While all three studies investigated balance-related tasks, none truly simulated free-standing balance in a supine condition.

The free-standing inverted pendulum model used in our simulator design distinguishes it from the previously developed systems. It allows for the tasks performed in the simulator to closely mimic free-standing balance with sensory feedback of relevant joints and muscle receptors, and motor pathways controlling tonic and reflexive muscle responses. In addition, the simulator load stiffness levels approached those seen during normal upright standing. Therefore, our fully MRI compatible simulator allows for functional neuroimaging to be combined with balance-relevant tasks to investigate the neural substrates of free-standing static and dynamic balance control.

Currently severely affected PD patients, and other clinical populations, in whom upright standing trials can no longer be safely carried out and/or that cannot stand unassisted for long enough periods of time are usually excluded from participating in postural instability research. The simulator could provide an opportunity to include these more severely-affected individuals as it would not require upright stance. The inclusion of these individuals would lead to postural instability research results that are more generalizable to the entire PD population.

There are a few limitations of the proposed simulator. First, as participants are supine, loading of the body and vestibular input is different compared to upright standing. Tightly fastened straps wrapped around the waist and shoulders were used to mimic, as much as possible, gravitational pull on the body, and resultant input from joint, Golgi tendon organ, and foot sole cutaneous receptors. While vestibular input was different, the fact that similar balance deficits with PD were seen between real and simulated balance tasks suggests a non-vestibular origin of the balance deficits. Second, it is not possible to assess balance behavior in the medial-lateral (ML) direction using the simulator in its current form. Prior studies have found ML sway to also be affected in PD patients (5, 6), with larger ML sway amplitude in PD patients. However, ML instability is generally much less pronounced, as reflected by the normally narrow-based gait and intact tandem gait test in even advanced PD stages (29, 30). Additionally, although different muscle/joints are involved in controlling AP and ML sway, there is no evidence to suggest they are controlled using different cortical or subcortical structures. Third, dyskinesia may prevent proper placement of the feet on the footplate of the simulator in PD patients. Therefore, further testing is needed to determine the feasibility of using the simulator in PD patients suffering from dyskinesia and other symptoms not apparent in this study.

In conclusion, the results of this study indicate that deficits in both static and dynamic balance control in PD_ON_ patients can be detected in recumbent individuals using a novel MRI compatible balance simulator. The simulator provides a unique opportunity to combine functional neuroimaging with balance-relevant tasks, and a new means to create insights into the cortical and subcortical structures contributing to postural instability in PD.

## Data Availability

The raw data supporting the conclusions of this manuscript will be made available by the authors, without undue reservation, to any qualified researcher.

## Ethics Statement

This study was carried out in accordance with the recommendations of the Clinical Research Ethics Board of the University of British Columbia with written informed consent from all subjects. All subjects gave written informed consent in accordance with the Declaration of Helsinki. The protocol was approved by the Clinical Research Ethics Board of the University of British Columbia.

## Author Contributions

EP, MM, BB, JI, and MC developed the study concept. EP, MM, TC, and MC contributed to organizing data collection. EP, TC, and MC contributed to data collection and design of the statistical analyses. EP and TC performed the statistical analyses. EP wrote the first draft of the manuscript. MM, TC, BB, JI, and MC reviewed and critiqued the statistical analyses and manuscript.

### Conflict of Interest Statement

The authors declare that the research was conducted in the absence of any commercial or financial relationships that could be construed as a potential conflict of interest.
