# Supplementary material for: A Novel MRI Compatible Balance Simulator to Detect Postural Instability in Parkinson's Disease
Source: Front Neurol. 2019 Aug 28;10:922. doi: 10.3389/fneur.2019.00922 (PMC6722178; doi:10.3389/fneur.2019.00922)
Supplement: Supplementary file 1 [file Data_Sheet_1.docx]

**Appendix 1:** Clinical characteristics Parkinson’s disease patients for study 1 and study 2

PD_ON_, Parkinson’s disease patients; Maximum Unified Parkinson’s Disease Rating Scale motor examination (UPDRS-ME) score is 108; Maximum Hoehn & Yahr (H&Y) score is 5.

| PD_ON_ | Age (years) | Disease duration (years) | UPDRS-ME score | | Hoehn & Yahr score | | Levodopa equivalent dose | | Antiparkinson medication |
| --- | --- | --- | --- | --- | --- | --- | --- | --- | --- |
| Study 1 | | | | | | | | | |
| 1 | 68 | 9 | 25 | 2 | | 900 | | Levodopa/carbidopa | |
| 2 | 76 | 10 | 38 | 3 | | 1638 | | Levodopa/carbidopa, Entacapone, Ropinirol | |
| 3 | 66 | 6 | 18 | 2 | | 682 | | Levodopa/carbidopa, Entacapone | |
| 4 | 78 | 1 | 28 | 2 | | 300 | | Levodopa/carbidopa | |
| 5 | 62 | 3 | 37 | 2 | | 250 | | Rasagiline, Pramipexole | |
| 6 | 73 | 3 | 36 | 3 | | 325 | | Levodopa/carbidopa, Rasagiline | |
| 7 | 68 | 8 | 46 | 3 | | 1100 | | Levodopa/carbidopa | |
| 8 | 65 | 4 | 38 | 3 | | 100 | | Rasagiline | |
| 9 | 64 | 11 | 21 | 2 | | 720 | | Levodopa/carbidopa, Rasagiline, Ropinirol | |
| 10 | 74 | 17 | 32 | 3 | | 1499 | | Levodopa/carbidopa, Entacapone, Amantadine | |
| 11 | 74 | 2 | 24 | 2 | | 550 | | Levodopa/carbidopa, Rasagiline | |
| 12 | 66 | 2 | 39 | 2 | | 800 | | Levodopa/carbidopa | |
| 13 | 75 | 2 | 14 | 2 | | 500 | | Levodopa/carbidopa | |
| 14 | 57 | 6 | 16 | 1 | | 500 | | Levodopa/carbidopa | |
| Range | **57-78** | **1-17** | **14-46** | **1-3** | | **100-1638** | |  | |
| Mean (SE) | **69.0 (1.6)** | **6.0**  **(1.2)** | **29.4**  **(2.7)** | **-** | | **704.6**  **(121.7)** | |  | |
| Study 2 | | | | | | | | | |
| 1 | 68 | 2 | 8 | 1 | | 225 | | Levodopa/carbidopa | |
| 2 | 72 | 2 | 31 | 2 | | 300 | | Levodopa/carbidopa | |
| 3 | 74 | 4 | 25 | 2 | | 325 | | Levodopa/carbidopa, Rasagiline | |
| 4 | 67 | 3 | 44 | 2 | | 850 | | Levodopa/carbidopa | |
| 5 | 73 | 6 | 36 | 2 | | 900 | | Levodopa/carbidopa, Rasagiline, Pramipexole | |
| 6 | 60 | 2 | 7 | 1 | | 300 | | Levodopa/carbidopa | |
| 7 | 67 | 3 | 25 | 2 | | 750 | | Levodopa/carbidopa | |
| 8 | 61 | 9 | 18 | 2 | | 750 | | Levodopa/carbidopa | |
| 9 | 64 | 14 | 46 | 3 | | 1150 | | Levodopa/carbidopa, Pramipexole | |
| 10 | 75 | 4 | 31 | 2 | | 775 | | Levodopa/carbidopa, Rasagiline | |
| 11 | 70 | 6 | 34 | 2 | | 575 | | Levodopa/carbidopa, Rasagiline | |
| 12 | 70 | 7 | 31 | 3 | | 1450 | | Levodopa/carbidopa, Rasagiline, Amantadine | |
| 13 | 65 | 3 | 27 | 2 | | 1360 | | Levodopa/carbidopa, Rotigotine | |
| 14 | 68 | 10 | 20 | 2 | | 900 | | Levodopa/carbidopa | |
| 15 | 59 | 7 | 19 | 1 | | 450 | | Levodopa/carbidopa | |
| 16 | 68 | 6 | 20 | 2 | | 948 | | Levodopa/carbidopa, Entacapone | |
| 17 | 69 | 1 | 34 | 2 | | 150 | | Levodopa/carbidopa | |
| 18 | 65 | 12 | 16 | 2 | | 1015 | | Levodopa/carbidopa, Rasagiline, Ropinirol | |
| 19 | 69 | 4 | 28 | 2 | | 200 | | Levodopa/carbidopa | |
| 20 | 67 | 6 | 39 | 2 | | 600 | | Levodopa/carbidopa, Rasagiline | |
| Range | **59-75** | **1-14** | **7-46** | **1-3** | | **150-1450** | |  | |
| Mean  (SE) | **67.6 (1.0)** | **5.6**  **(0.8)** | **27.0**  **(2.4)** | **-** | | **698.7**  **(85.9)** | |  | |

**Appendix 2:** Results static and dynamic balancing tasks for study 1 and study 2

Data are displayed as mean (SE) for analysis of variance (study 1), and mean (SE) and median for independent t-tests and Mann-Whitney tests (study 2). PD_ON_, Parkinson’s disease patients; AP, anterior-posterior; RMS, root mean square; MPF, mean power of frequency; F_(x,x)_, F-value_(degrees of freedom)_; 𝜂_p_^2^, partial eta-squared; U, Mann-Whitney U statistic; z, *z*-score; t(x), *t*-statistic(degrees of freedom); *d*, Cohen’s *d*; 𝜂^2^, eta-squared; ^+^ Data were log-transformed, for ease of interpretation mean (SE) values of the non-log-transformed data are shown; *p* < 0.05 shown in bold.

|  | Real balance | | | Simulated balance | | |
| --- | --- | --- | --- | --- | --- | --- |
|  | **Controls** | **PD_ON_** | **Statistics** | **Controls** | **PD_ON_** | **Statistics** |
| Study 1 – Static balancing | | | | | | |
| AP-RMS (°)  *Mean*  *(SE)* | 0.445 (0.036) | 0.557 (0.043) | F_(1,32)_ = 4.725  ***p* = 0.037^+^**  𝜂_p_^2^ = 0.129 | 0.521 (0.037) | 0.676 (0.044) | F_(1,32)_ = 7.205  ***p* = 0.011**  𝜂_p_^2^ = 0.184 |
| AP-MPF (Hz)  *Mean*  *(SE)* | 0.113 (0.010) | 0.113 (0.012) | F_(1,32)_ = 0.000  *p* = 0.984  𝜂_p_^2^ = 0.000 | 0.125 (0.010) | 0.103 (0.012) | F_(1,32)_ = 2.059  *p* = 0.161^+^  𝜂_p_^2^ = 0.060 |
| Study 2 – Static balancing | | | | | | |
| AP-RMS (°)  *Mean*  *(SE)*  *Median* | 0.347  (0.035)  0.331 | 0.421  (0.029)  0.438 | U = 132.0,  z = -2.034  ***p* = 0.042**  𝜂^2^ = 0.106 | 0.518  (0.045)  0.500 | 0.851  (0.113)  0.738 | U = 108.0,  z = -2.660  ***p* = 0.007**  𝜂^2^ = 0.181 |
| AP-MPF (Hz)  *Mean*  *(SE)*  *Median* | 0.108 (0.009)  0.115 | 0.095 (0.006)  0.093 | t(39) = 1.235  *p* = 0.224  *d* = 0.386 | 0.079  (0.013)  0.054 | 0.080  (0.009)  0.067 | U = 177.0,  z = -0.861  *p* = 0.401  𝜂^2^ = 0.019 |
| Study 2 – Dynamic balancing | | | | | | |
| Peak displacement (°)  *Mean*  *(SE)*  *Median* | 2.535 (0.112)  2.462 | 2.705 (0.137)  2.646 | t(27) = -0.965  *p* = 0.343  *d* = -0.359 | 3.306  (0.154)  3.046 | 3.930  (0.185)  3.954 | U = 55.0,  z = -2.182  ***p* = 0.029**  𝜂^2^ = 0.170 |
| Peak velocity (°/s)  *Mean*  *(SE)*  *Median* | 9.271 (0.432)  9.152 | 9.395 (0.507)  9.101 | t(27) = -0.187  *p* = 0.853  *d* = -0.070 | 8.842  (0.349)  8.676 | 9.511  (0.378)  9.106 | U = 78.0,  z = -1.178  *p* = 0.252  𝜂^2^ = 0.050 |
| TTP displacement (s)  *Mean*  *(SE)*  *Median* | 0.619  (0.020)  0.626 | 0.649  (0.027)  0.610 | U = 91.0,  z = -0.611  *p* = 0.561  𝜂^2^ = 0.013 | 0.721 (0.024)  0.728 | 0.843 (0.046)  0.814 | t(19.789) = -2.344  ***p* = 0.030**  *d* = -0.871 |
| TTP velocity (s)  *Mean*  *(SE)*  *Median* | 0.283 (0.013)  0.283 | 0.284 (0.008)  0.287 | t(27) = -0.069  *p* = 0.945  *d* = -0.026 | 0.313  (0.007)  0.308 | 0.336  (0.008)  0.330 | U = 34.0,  z = -3.099  ***p* = 0.001**  𝜂^2^ = 0.343 |
